# A modeling study of the Danish HIV epidemic in men who have sex with men: travel, pre-exposure prophylaxis and elimination

**DOI:** 10.1038/s41598-018-33570-0

**Published:** 2018-10-30

**Authors:** Laurence Palk, Jan Gerstoft, Niels Obel, Sally Blower

**Affiliations:** 1 Center for Biomedical Modeling, Semel Institute for Neuroscience and Human Behavior, David Geffen School of Medicine, University of California, Los Angeles, CA 90024 USA; 2 Department of Infectious Diseases, Copenhagen University Hospital, Rigshospitalet, Denmark

## Abstract

UNAIDS has identified the Danish HIV epidemic in men who have sex with men (MSM) as a priority for elimination. Incidence is close to the elimination threshold of one new infection per year per 1,000 individuals. However, surveillance data show that HIV strains are being imported into Denmark, mainly due to travel. We use a transmission model to predict (from 2018 to 2030) the impact of pre-exposure prophylaxis (PrEP) on incidence. Our model reflects the current epidemic and diagnosis rates in the Danish MSM community. We conduct a sensitivity analysis based on 20,000 simulations, and assume that PrEP coverage could range from zero to 50% and diagnosis rates increase up to three-fold. We predict that incidence will fall below the elimination threshold, even without the introduction of PrEP, reaching 0.87 (median, 95% Confidence Interval: 0.65–1.23) new infections per 1,000 MSM by 2030. PrEP could reduce incidence to well below the threshold, if it results in a significant increase in diagnosis rates and reduces the number of infections occurring abroad. The Danish Medicine Agency and Danish Health Authority have recommended introducing PrEP. Our study provides strong support for this recommendation, and shows the importance of Danish MSM using PrEP when abroad.

## Introduction

The Danish HIV epidemic in men who have sex with men (MSM), an epidemic UNAIDS has identified as a priority for elimination^1^, is approaching the WHO elimination threshold of one new HIV infection per year per 1,000 individuals^2^. We have shown previously that incidence has continuously decreased over the past two decades as a result of a high coverage of treatment^2^. These results are based on data from the Danish HIV Cohort Study (DHCS), an ongoing nationwide population-based study^3,4^. By 2013, the vast majority (~80%) of MSM in Denmark who were infected with HIV were on treatment^2^. Our study provided proof-of-concept that “treatment as prevention” (TasP), given high adherence, can substantially reduce a country’s HIV epidemic. Recently the Danish Medicine Agency and the Danish Health Authority have recommended that pre-exposure prophylaxis (PrEP) should be used as an additional prevention modality, but it has not yet been rolled out. Randomized controlled studies have demonstrated that PrEP is highly effective in preventing infection with HIV^5^. PrEP is already being used in the United States and a number of European countries^6^. Here we predict the potential impact of PrEP on the HIV epidemic in MSM in Denmark as incidence approaches the elimination threshold. Our predictions run from 2018 to 2030. Introducing PrEP may increase diagnosis rates, as individuals will be tested for HIV before they are provided with PrEP. Consequently, we predict the impact of PrEP in both the presence and absence of increasing diagnosis rates.

As transmission within a country decreases to very low levels it is important to consider the importation of infections from outside the country. Multiple phylogenetic studies^7–10^ have shown that national borders do not contain the spread of HIV. Danish surveillance data show that strains from other countries have been, and continue to be, imported into Denmark: ~13% of new HIV infections in Danish MSM are acquired when travelling abroad^11^. Strain importation can potentially decrease the probability of eliminating HIV. Therefore, to make predictions of the impact of PrEP in Denmark we designed a parsimonious within-country transmission model that includes the potential for strain importation. The model includes two pathways: non-residents (infected with HIV) can enter Denmark, and residents can become infected whilst travelling abroad.

The Danish MSM, whom were assumed to have been infected abroad, had visited other European countries and/or the United States^11^. Prevalence of HIV, treatment coverage, and condom usage varies substantially both among, and within, communities of MSM in Europe and the United States^12,13^. However in all of these communities, HIV prevalence is higher and treatment coverage lower, than in the MSM community in Denmark^12,13^. Denmark has been shown to have been one of the most successful countries in the world in reducing their HIV epidemic, and was already close to the elimination threshold by 2016^2^.

Denmark is exceptional with regard to HIV healthcare. Diagnosis rates are extremely high and almost all diagnosed individuals are linked to care and begin treatment (on average) in less than two weeks^14^. In addition, 98% of patients are fully adherent to treatment and virally suppressed. In Denmark, as in many other countries, all HIV-infected individuals are eligible for treatment regardless of their CD4 cell count.

## Methods

**Figure 1:**
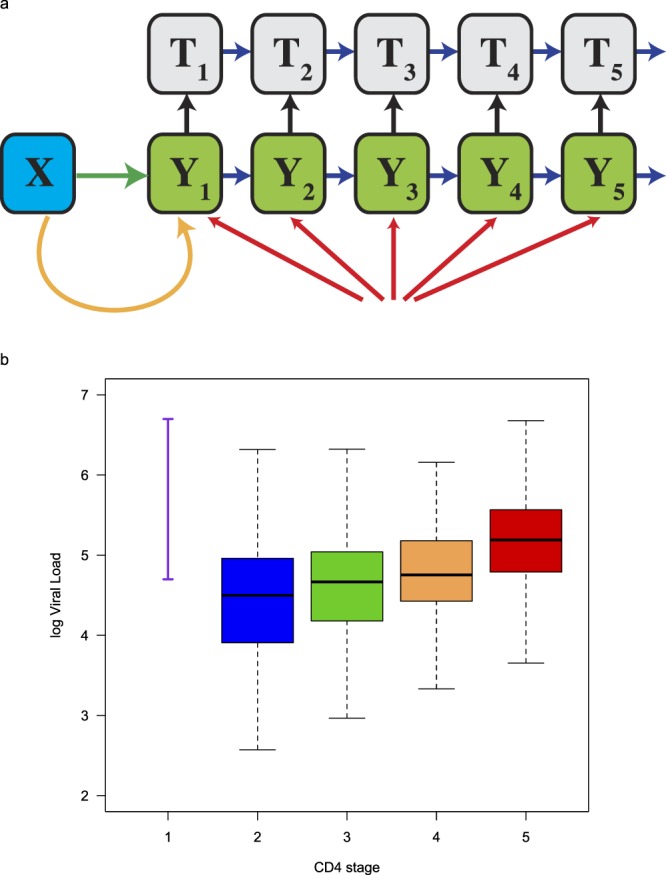
Model structure and viral load data used for parameterization. (**a**) The transmission model includes susceptible MSM (*X*), infected undiagnosed MSM (*Y*), and MSM on treatment (*T*). The green arrow represents infections occurring in Denmark. The orange arrow represents infections occurring abroad. Red arrows represent non-resident MSM arriving infected with HIV. Blue arrows represent disease progression. Black arrows represent diagnosis and linkage to treatment. The model is described in detail in the SM. (**b**) Viral load data used to calculate the infectivity of individuals in each stage of the model. Data for primary infection (represented by the purple line) were taken from the literature; viral load data for the other four stages of infection (represented by the blue, green, orange and red data) are from the DHCS.

A schematic of the transmission model is shown in Fig. 1a. Undiagnosed HIV-infected MSM are grouped into five stages of infection, stratified on the basis of CD4 cell counts; CD4 cell counts in treated individuals are not tracked. The model structure reflects the current recommendations for treatment in Denmark: i.e., individuals in any stage of infection can be treated. In the model, treated individuals differ from untreated individuals in two respects: (i) they are substantially less infectious (due to a treatment-induced reduction in viral load), and (ii) their life expectancy is considerably longer (due to a treatment-induced reduction in the disease progression rate). A more detailed description of the model, including equations, is given in section 1 of the Supplementary Material (SM).

Section 2 of the SM contains a description of how the rollout of PrEP was modeled; equations are presented. The structure of the model allows for PrEP to be used by uninfected MSM both in Denmark and when travelling abroad. PrEP coverage is defined as the percentage of uninfected MSM who use PrEP. We assume, that in general, the same percentage of MSM who use PrEP in Denmark will use PrEP when abroad, and that PrEP reduces the risk of infection by 60%^5^. We also considered the potential impact of future increases in diagnosis rates that may accompany the rollout of PrEP. Notably, due the excellent linkage to care in Denmark, increases in diagnosis rates would directly (and very quickly) translate into increases in treatment rates.

The model was parameterized to reflect the current epidemiological conditions and treatment programs in the MSM community in Denmark, see section 3 in the SM. We used viral load data (shown by the purple line in Fig. 1b) and an empirically derived function^15–17^ to calculate the infectivity of an individual in primary infection: stage one of the model represents primary infection. The viral load data were taken from the literature^18,19^. We calculated the infectivity of undiagnosed individuals in stages two to five of the model relative to the infectivity of an individual in primary infection. The viral load data used to make these calculations are shown in the form of boxplots in Fig. 1b; blue (stage 2), green (stage 3), orange (stage 4), and red (stage 5). These data had been collected from 1,059 diagnosed treatment-naïve MSM who participated in the DHCS. Values for all model parameters are given in Table S1 in the SM.

The model was simulated 20,000 times in MATLAB^20^ using a Markov Chain Monte Carlo (MCMC) toolbox after an initial burn-in of 1,000 simulations. To set the initial conditions, we used treatment data from the DHCS and estimates of the number of undiagnosed individuals that we had determined (using DHCS data) in a previous study^2^. We calibrated the model by beginning each of the simulations in 2007 and fitting the data to a decade of data from several sources: DHCS data on HIV diagnosis rates (stratified into four stages based on CD4 cell counts), DHCS data on the number of MSM on treatment, surveillance data on the annual number of non-resident MSM who arrive in Denmark infected with HIV, and surveillance data on the annual number of Danish MSM who become infected abroad. Each year, approximately 12 Danish MSM become infected with HIV when travelling abroad^11^. A more detailed description of the calibration methodology is given in section 4 in the SM.

We predicted the impact of a rollout of PrEP, and an increase in diagnosis rates, on the number of newly acquired infections between 2018 and 2030. We conducted a sensitivity analysis and investigated a wide range of conditions: coverage ranging from zero to 50%, and diagnosis rates (which are already high) increasing up to three-fold. We investigated all possible combinations of these conditions. In addition, we conducted a scenario analysis based on differential PrEP usage; we assumed that PrEP would be used more by MSM who travel than by those who don’t travel.

### Ethics Approval

No approval from an ethics committee was necessary as we conducted an analysis of a dataset that does not include identifiers.

## Results

**Figure 2:**
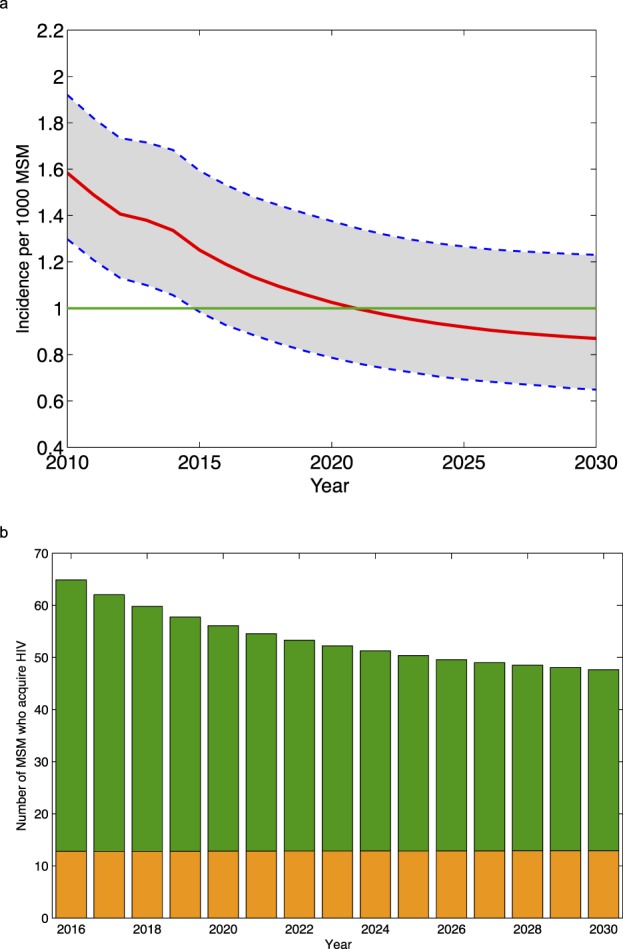
Predictions made under the assumption that diagnosis rates do not change and PrEP is not introduced. (**a**) Predictions for incidence (in terms of the number of new HIV infections per 1,000 MSM). The red line shows the median value and the shaded grey area the 95% CI of 20,000 simulations. The green line shows the WHO elimination threshold. (**b**) Predictions for the number of MSM who acquire HIV each year. Infections shown in green occur in Denmark. Infections shown in orange occur abroad.

Figure 2a shows the predicted trend in incidence, under the assumption that diagnosis rates (and hence treatment rates) remain at their current level and PrEP is not introduced. Predictions are based on 20,000 simulations. Notably, even under current conditions, we predict that the annual incidence rate will fall below the WHO elimination threshold. We predict, that by 2030, the annual incidence will have fallen to a low of 0.87 (median value, 95% Confidence Interval, CI: 0.65–1.23) new HIV infections per 1,000 MSM. Notably, our modeling shows that as treatment coverage continues to increase, the rate of decline in the incidence rate will decrease and incidence will stabilize by ~2025; we refer to this as a TasP-saturation effect.

Figure 2b shows predictions for the temporal trend in the number of MSM who are Danish residents who acquire HIV each year. The result from the most likely scenario of the 20,000 simulations is shown; this scenario was identified using the likelihood function. Not surprisingly, as incidence decreases, the number of MSM whom become infected abroad each year becomes an increasing fraction of the total number of new infections, Fig. 2b.

**Figure 3:**
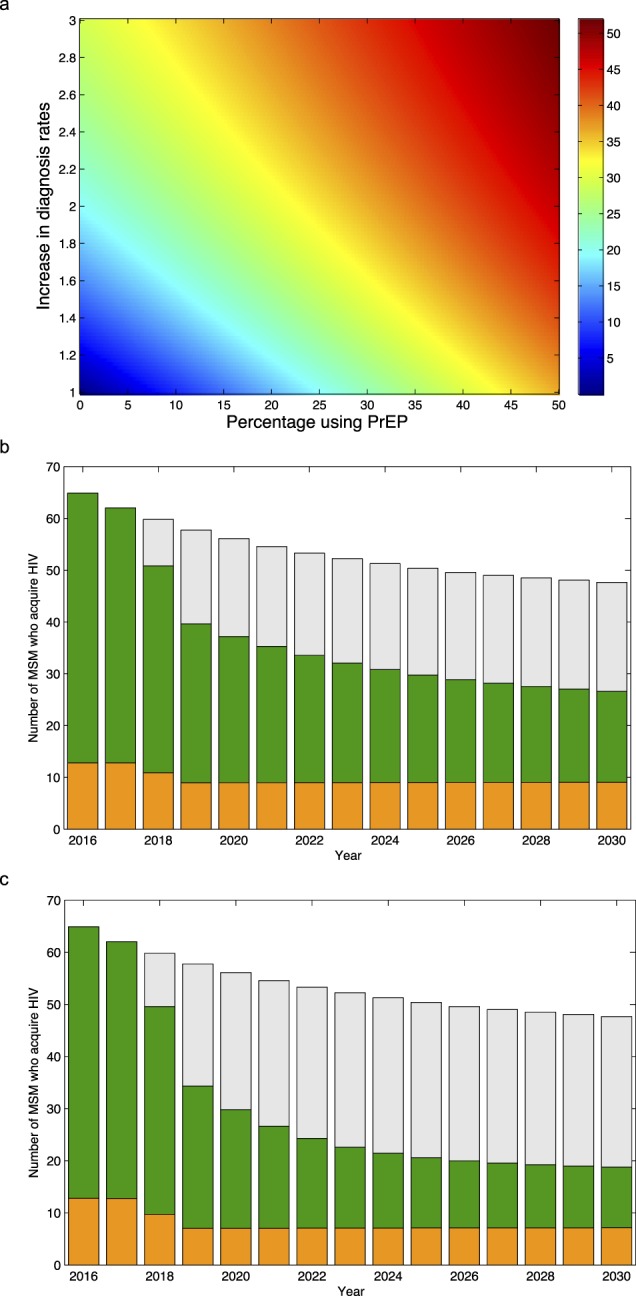
Predictions showing the impact of introducing PrEP with, and without, an increase in diagnosis rates. (**a**) Heat map showing results from the sensitivity analysis: predictions for the effectiveness of PrEP with, and without, an increase in diagnosis (and hence treatment) rates. The magnitude of the increase in the diagnosis rate in each CD4 stage is relative to the current diagnosis rate. The color scale shows effectiveness as a percentage reduction in the cumulative number of infections between 2018 and 2030. (**b**) Predictions for the number of MSM who acquire HIV each year under the assumption that PrEP coverage reaches 50%, but diagnosis rates remain at their current levels. Infections shown in green occur in Denmark. Infections shown in orange occur abroad. Grey bars show predicted values without PrEP. (**c**) Predictions for the number of MSM who acquire HIV each year under the assumption that the introduction of PrEP will be accompanied by a doubling of diagnosis rates, PrEP coverage in Denmark reaches 50%, and 80% of Danish MSM use PrEP when they travel abroad. Infections shown in green occur in Denmark. Infections shown in orange occur abroad. Grey bars show predicted values without PrEP or an increase in diagnosis rates.

The results from the sensitivity analysis are presented in the form of a heat map (Fig. 3a). Results show, for each combination of parameter values, the predicted effectiveness of PrEP. Effectiveness is defined in terms of the cumulative number of infections that would be prevented if conditions change (i.e., PrEP is introduced) relative to the cumulative number of infections that would occur if conditions remain the same; i.e., diagnosis rates remain at their current level and PrEP is not introduced. Effectiveness is calculated over the time period 2018 to 2030. The effectiveness of PrEP, in the absence of any increase in diagnosis rates, could be as high as 45% (Fig. 3a); however, this would only occur if ~50% of uninfected MSM used PrEP when they engaged in risky sexual behavior. Notably, if substantial increases in diagnosis rates accompanied the rollout, the effectiveness of PrEP could be extremely high even if coverage was fairly low (Fig. 3a). For example: if coverage was only 15% and diagnosis rates doubled, ~25% of HIV infections would be prevented by 2030.

Importantly, the heat map shows that very different conditions can prevent the same number of infections; where conditions are defined in terms of PrEP coverage and diagnosis rates. Conditions are equivalent, in terms of effectiveness, if they occur in the same color zone (Fig. 3a). For example, many different conditions could reduce the cumulative incidence by ~30%; any combination of parameter values that lie along, or close to, the yellow line in the heat map would cause this magnitude of reduction. This includes, for example: a coverage of PrEP of ~45% with no change in diagnosis rates, a coverage of ~5% with a tripling of diagnosis rates, and a coverage of 25% with an 80% increase in diagnosis rates.

Introducing PrEP - even if it is not accompanied by an increase in diagnosis rates - could be very effective, as it would reduce transmission within Denmark and also the number of infections that occur abroad. For example, achieving a PrEP coverage of 50% would reduce the number of MSM who become infected in Denmark (between 2018 and 2030) by 40% and the number of MSM who become infected abroad by ~30% (Fig. 3b). Significantly more infections would be prevented - but only within Denmark - if an increase in diagnosis rates accompanied the rollout of PrEP. The effectiveness of PrEP could be further increased if there was a differential usage of PrEP between travelers and non-travelers. A higher usage of PrEP among MSM who travel abroad could reduce incidence in Denmark to well below the elimination threshold (Fig. 3c).

## Discussion

We have previously shown that TasP had reduced incidence in Danish MSM close to the WHO elimination threshold by 2013^2^. Here, using data from the DHCS and a transmission model, we predict that without any change in current conditions, incidence in Danish MSM will fall below the elimination threshold before 2030. However, we have also found a TasP-saturation effect can be expected and - if conditions remain the same - the incidence rate is likely to stabilize. This is because treatment coverage and population-level viral suppression have already reached very high levels, and a TasP-induced reduction in incidence has been occurring for approximately two decades^2^. As incidence declines, an increasing fraction of new infections will be acquired abroad. Importantly, our results show that introducing PrEP to Denmark could substantially reduce incidence to well below the elimination threshold. This could occur - even if PrEP coverage is only low to moderate - if significant increases in diagnosis rates accompany the rollout of PrEP and if it is used by MSM when traveling abroad.

Previous modeling studies have predicted the effects of PrEP or TasP on reducing HIV epidemics^2,21–25^. However, these studies have been based on the implicit assumption that epidemics occur in closed populations. They have not considered the effect of interventions on reducing HIV epidemics when infections can be acquired abroad. TasP alone can only reduce transmission within a country. However, PrEP is capable of reducing transmission within a country and also reducing the number of infections acquired abroad. PrEP is also likely to be accompanied by an increase in diagnosis rates as individuals will need to be tested before they are prescribed PrEP. Consequently, rolling out PrEP is likely to both directly and indirectly prevent infections: uninfected individuals who take PrEP will have their risk of acquiring HIV reduced, HIV-infected individuals who are diagnosed before being prescribed PrEP will be put on treatment and contribute to a TasP-induced reduction in the incidence rate.

As with all studies, ours has limitations. We have not explicitly modeled HIV epidemics in countries that are linked through travel to Denmark. As treatment coverage is expanded in these countries the risk of infection for Danish MSM whilst travelling abroad is likely to decrease. However, this is unlikely to occur quickly; no other country has a treatment coverage and viral suppression rate that is as high as in Denmark. Risk behaviors may decrease or increase, both within Denmark and abroad. If risk behavior increases substantially, it may slow the reduction in the incidence rate; conversely, if risk behavior decreases substantially, it will shorten the time to reach the elimination threshold. We have not modeled changes in risk behaviors, because it would have required making many unverifiable assumptions. For the same reason, we have not modeled HIV co-infection with other sexually transmitted diseases; infection with another sexually transmitted disease increases the risk of infection with HIV, co-infection increases infectivity^26,27^. We have used a fairly parsimonious model that could easily be modified to increase its structural complexity, for example, by including age structure or behavioral heterogeneity. However, parsimonious models have been shown to provide considerable insight^28,29^. Notably - since a fairly high fraction of infections occur in Danish MSM who travel abroad - promoting a very high usage of PrEP in travelers could be an effective, and feasible, means to target “high-risk” MSM for prevention.

PrEP has recently been introduced in several European countries: England, Scotland, Norway, Belgium, France and Portugal^6^. Considerable demand for PrEP exists in Denmark, as demonstrated by the rapid enrolment of Danish MSM in the DISCOVER trial; a trial to test the efficacy of a new PrEP formulation, emtricitabine and tenofovir alafenamide (F/TAF)^30^. The Danish Medicine Agency and the Danish Health Authority have recommended that PrEP should be rolled out, but it is not clear when this will occur. Currently, it is only available in Denmark to those who can afford to buy their drugs abroad or via the internet, or to patients who are referred to infectious disease specialists. PrEP, used by MSM, has recently been shown to be cost-effective^22^, and Truvada^TM^ (the first PrEP drug to be approved) has dramatically decreased in price as a generic version has become available. When PrEP is rolled out it will be important to monitor for risk compensation. If this occurs it could lead to an increase in the incidence of HIV and other sexually transmitted diseases; the incidence of gonorrhea and syphilis in Danish MSM has been rising^31,32^. Studies conducted to date have been inconclusive in determining whether risk compensation occurs due to PrEP usage^33^. Regardless, it will be essential to encourage MSM to use condoms and reduce risky behaviors. Our study provides strong support for providing PrEP to MSM in Denmark and increasing diagnosis rates. Taken together, our results demonstrate that if Danish MSM use PrEP, especially when travelling abroad, it could be possible to reduce HIV incidence to well below the elimination threshold by 2030. Denmark would be the first country to reduce HIV incidence to such an extremely low level. If this occurs, it could provide guidance on effective prevention strategies for other countries with HIV epidemics that UNAIDS has prioritized for elimination^1^.

## Electronic supplementary material

Supplementary Material

## Data Availability

The data supporting the findings of this study are available from the corresponding author upon reasonable request.
