# Supplementary material for: A modeling study of the Danish HIV epidemic in men who have sex with men: travel, pre-exposure prophylaxis and elimination
Source: Sci Rep. 2018 Oct 30;8:16003. doi: 10.1038/s41598-018-33570-0 (PMC6207710; doi:10.1038/s41598-018-33570-0)

SUPPLEMENTARY MATERIAL

A modeling study of the Danish HIV epidemic in men who have sex with men: travel, pre-exposure prophylaxis and elimination

Laurence Palk,[1] Jan Gerstoft,[2] Niels Obel,[2] Sally Blower[1*]

[1]Center for Biomedical Modeling, Semel Institute for Neuroscience and Human Behavior, David Geffen School of Medicine, University of California, Los Angeles, CA 90024.

[2]Department of Infectious Diseases, Copenhagen University Hospital, Rigshospitalet, Denmark.

*Corresponding author

1. Model Structure

The transmission dynamic model is designed to reflect the HIV epidemic in the MSM community in Denmark. The model includes susceptible MSM (*X*), infected undiagnosed MSM (*Y*), and MSM on treatment (*T*). Undiagnosed MSM are grouped into five stages of infection that are specified in terms of CD4 cell counts and viral load. The first stage (*i*=1), primary infection, is short in duration but has a very high infectivity due to high viral load[18, 19]; it includes individuals with CD4 counts of >900 cells/μL. Stage two (*i*=2) includes individuals with CD4 counts of 500-900 cells/μL. Stages three to five (*i*=3,4,5) include individuals with CD4 counts of 350-500 cells/μL, 200-350 cells/μL, <200 cells/μL, respectively. Treated MSM are grouped into five sequential stages that correspond to the CD4 cell stage an individual is in when they initiate treatment; however, no assumptions are made regarding how CD4 counts change during treatment. The model is defined using 11 ordinary differential equations.

The change in the number of susceptible individuals over time is given by:

$$\frac{dX}{dt} = r - \mu X - \frac{cX}{N}\left(\sum_i \beta_i Y_i + \delta\beta_i T_i\right) - \alpha X$$

where *r* is the recruitment rate into the MSM community (i.e., the number of uninfected MSM who join the community each year) and *μ* is the per capita rate at which MSM leave the population through death or emigration. *c* is the transmission rate for primary infection; it is the product of the average number of sex partners per year and the probability of HIV transmission per partnership, given the partner is in primary infection. β_i is the CD4 stage-specific relative transmissibility and is calculated from the DHCS data; it expresses the relative rate of transmission in each stage, relative to the transmission rate in primary infection. *δ* is the relative infectivity of treated individuals compared to untreated, α is the per capita probability of travelling and becoming infected abroad, and *N* is the total size of the MSM community:

$$N = X + \sum_i Y_i + \sum_i T_i \ .$$

The change in the number of infected undiagnosed individuals over time is given by the following five equations. Individuals in primary infection are specified by Y_1, individuals in the subsequent four stages by Y_i:

$$\frac{dY_1}{dt} = \frac{cX}{N}\left(\sum_i \beta_i Y_i + \delta\beta_i T_i\right) + \alpha X + \lambda_1 - p_1 Y_1 - d_1 Y_1 - \mu Y_1$$

$$\left.\frac{dY_i}{dt}\right|_{i=2...5} = p_{i-1} Y_{i-1} + \lambda_i - d_i Y_i - \mu Y_i$$

where p_i is the per capita disease progression rate for stages one to four and the per capita death rate for stage five, and d_i is the per capita diagnosis rate for each of the five stages. The rate (i.e., number per year) of non-resident MSM who arrive in Denmark in each stage of HIV infection i, is given by λ_i. The total number of non-residents who arrive infected each year is equal to $\sum \lambda_i = \Lambda$. The ratio of the number who arrive in each stage of infection matches the steady-state number of individuals in each stage, i.e.

$$\frac{\lambda_i}{\Lambda} = \frac{1/p_i}{\sum_i \left(1/p_i\right)}$$

Individuals on treatment are divided into five corresponding stages:

$$\frac{dT_1}{dt} = d_1 Y_1 - \phi p_1 T_1 - \mu T_1$$

$$\left.\frac{dT_i}{dt}\right|_{i=2..5} = d_i Y_i + \phi p_{i-1} T_{i-1} - \phi p_i T_i - \mu T_i$$

where Φ is the degree of treatment-induced reduction in the disease progression rate.

2. Modeling the rollout of PrEP

The rollout was modeled by adjusting the equations such that a fraction (Π) of the susceptible population have their risk of infection reduced by a factor *s*:

$$\frac{dX}{dt} = r - \mu X - \frac{cX}{N}\left(1 - \Pi + s\Pi\right)\left(\sum_i \beta_i Y_i + \delta \beta_i T_i\right) - \alpha X\left(1 - s\Pi\right)$$

$$\frac{dY_1}{dt} = \frac{cX}{N}\left(1 - \Pi + s\Pi\right)\left(\sum_i \beta_i Y_i + \delta \beta_i T_i\right) + \alpha X\left(1 - s\Pi\right) + \lambda_1 - p_1 Y_1 - d_1 Y_1 - \mu Y_1$$

$$\left.\frac{dY_i}{dt}\right|_{i=2...5} = p_{i-1} Y_{i-1} + \lambda_i - d_i Y_i - \mu Y_i$$

To model the roll out we set the PrEP coverage (i.e., the fraction of susceptible MSM using PrEP) in 2018 to be Π/2, and then increased it to Π from 2019 onwards.

We predicted the impact of PrEP in both the presence and absence of increasing diagnosis rates. We increased the values for the diagnosis rates for undiagnosed individuals in stages 1-4 relative to their current diagnosis rates. We did not increase the diagnosis rate in stage 5 as this rate is already very high.

3. Parameter Estimation

We estimated the relative transmission probability (β_i) for undiagnosed individuals in each of the five stages of the model, relative to the primary infection stage. We calculated stage-specific estimates for the

infectivity by using an empirically derived function[16, 17] and viral load data from diagnosed treatment-naïve MSM who participated in the DHCS: these data are shown in Fig 1B in the main text. The estimated values of the transmission probabilities are given in Table S1.

The per capita diagnosis rate (d_i) for individuals in each stage of the five stages of the model was estimated from the corresponding stage-specific diagnosis probability (δ_i), using the relationship $\delta_i = (1\text{-}\exp[-d_i])$. These diagnosis probabilities had been estimated in a previous study[2], using data from the DHCS. The diagnosis probability for individuals in stage one was assumed to be the same as in stage two. For 2014 onward the diagnosis rates were assumed to be constant and were set at the maximum rate estimated from a previous study[1] for the years 2007-2013 (values shown in Table S1).

We estimated values for four unknown parameters during the model calibration stage: the recruitment rate into the MSM community (r), the transmission rate for primary infection (c), the arrival rate (i.e., number per year) of non-resident HIV-infected MSM (Λ), and the per capita probability of travelling and becoming infected abroad (α).

All parameter estimates/values and their sources are given in Table S1.

4. Model calibration

The period from 2007 to 2013 was used for model calibration and for estimating values for the four unknown parameters: i.e., the recruitment rate into the MSM community (r), the transmission rate for primary infection (c), the arrival rate of non-resident HIV-infected MSM (Λ), and the per capita probability of travelling and becoming infected abroad (α).

We began with an initial burn-in of 1,000 simulations, then continued with 20,000 simulations. During the MCMC process the unknown parameters were sampled from uniform distributions with the following ranges: 2.4 to 24.2 for the transmission rate for primary infection; 200 to 1,600 for the recruitment rate; 0 to 15 for the arrival rate of non-resident HIV-infected MSM; and $1\text{x}10^{-6}$ to $1\text{x}10^{-3}$ for the per capita probability of getting infected when travelling abroad. In each simulation the values of each of the four parameters were updated according to a Metropolis-Hastings algorithm. Using a sum-of-squares likelihood function we compared the goodness-of-fit of the outputs of the model to data from the DHCS (that have been reported, or estimated, previously[2]) and surveillance data[11]: (i) the annual number of diagnoses stratified by CD4 stage, (ii) the total number of MSM on treatment, (iii) the annual incidence, (iv) the number of MSM who were infected when travelling abroad, and (v) the number of non-resident MSM who arrive infected with HIV. During the calibration period we included the constraint that in every simulation the size of the MSM community remained approximately constant. By the end of the calibration period the posterior distributions for the values of the four unknown parameters had converged to approximately normal distributions, Figure S1: values are given in Table S1.

Supplementary Material References

34. Wagner, B. G. & Blower, S. Universal access to HIV treatment versus universal 'test and treat': transmission, drug resistance & treatment costs. *PLoS ONE* **7**, e41212, https://doi.org/10.1371/journal.pone.0041212 (2012).

35. Time from HIV-1 seroconversion to AIDS and death before widespread use of highly-active antiretroviral therapy: a collaborative re-analysis. Collaborative Group on AIDS Incubation and HIV Survival including the CASCADE EU Concerted Action. Concerted Action on SeroConversion to AIDS and Death in Europe. *Lancet* **355**, 1131–1137 (2000).

36. Cohen, M. S. et al. Prevention of HIV-1 infection with early antiretroviral therapy. *N Engl J Med* **365**, 493–505, https://doi.org/10.1056/NEJMoa1105243 (2011).

37. Legarth, R. A. et al. Long-Term Mortality in HIV-Infected Individuals 50 Years or Older: A Nationwide, Population-Based Cohort Study. *J Acquir Immune Defic Syndr* **71**, 213–218, https://doi.org/10.1097/qai.0000000000000825 (2016).

38. Marcus, U., Hickson, F., Weatherburn, P. & Schmidt, A. J. Estimating the size of the MSM populations for 38 European countries by calculating the survey-surveillance discrepancies (SSD) between self-reported new HIV diagnoses from the European MSM internet survey (EMIS) and surveillance-reported HIV diagnoses among MSM in 2009. *BMC Public Health* **13**, 919, https://doi.org/10.1186/1471-2458-13-919 (2013).

Table S1: Parameters for the transmission dynamic model. N represents the Normal Distribution.

Parameter Description	Parameter symbol	Value	Source
**Per capita disease progression rates (1/years)**			
-Primary infection (stage 1, CD4>900)	p_1	**6**	Ref [34]
-Stage 2 (CD4 500-900)	p_2	**0.21**	Ref [35]
-Stage 3 (CD4 350-500)	p_3	**0.36**	Ref [35]
-Stage 4 (CD4 200-350)	p_4	**0.44**	Ref [35]
-Stage 5 (CD4 <200)	p_5	**0.33**	Ref [35]
**Relative transmissibility**			
-Primary infection (stage 1)	β_1	**1**	Estimated from Ref [18, 19]
-Stage 2	β_2	**0.294**	Estimated based on DHCS viral load data, see SM
-Stage 3	β_3	**0.351**	Estimated based on DHCS viral load data, see SM
-Stage 4	β_4	**0.351**	Estimated based on DHCS viral load data, see SM
-Stage 5	β_5	**0.571**	Estimated based on DHCS viral load data, see SM
**Per capita background death rate**	μ	**0.017**	
**Per capita diagnosis rates (1/years)**			
-Primary infection (stage 1)	d_1	**0.128**	Ref [2]
-Stage 2	d_2	**0.128**	Ref [2]
-Stage 3	d_3	**0.142**	Ref [2]
-Stage 4	d_4	**0.219**	Ref [2]
-Stage 5	d_5	**0.665**	Ref [2]
**Relative infectivity of treated individuals compared to untreated**	δ	**0.04**	Ref [36]
**Degree of treatment-induced reduction in disease progression**	Φ	**0.005**	Ref [37]
**PrEP risk reduction factor**	s	**0.4**	Ref [5]
**Size of MSM community**	N	**N(54700,4000)**	Ref [38]
**Transmission rate, primary infection**	c	**N(6.9,0.65)**	Posterior, estimated during model calibration, SM
**Recruitment rate (number per year)**	r	**N(951,107)**	Posterior, estimated during model calibration, see SM
**Number of MSMs (non-residents) who arrive infected with HIV (per year)**	Λ	**N(3.64,1.0)**	Posterior, estimated during model calibration, see SM
**Per capita probability of travelling and becoming infected abroad (per year)**	α	**N(2.5E-4,7.1E-5)**	Posterior, estimated during model calibration, see SM

Fig S1: Histograms showing convergence of the four unknown model parameters. The transmission rate for primary infection (*c*), the recruitment rate (per year) into the MSM community (*r*), the arrival rate (per year) of non-resident HIV-infected MSM (Λ), and the per capita probability of travelling and becoming infected abroad (α).

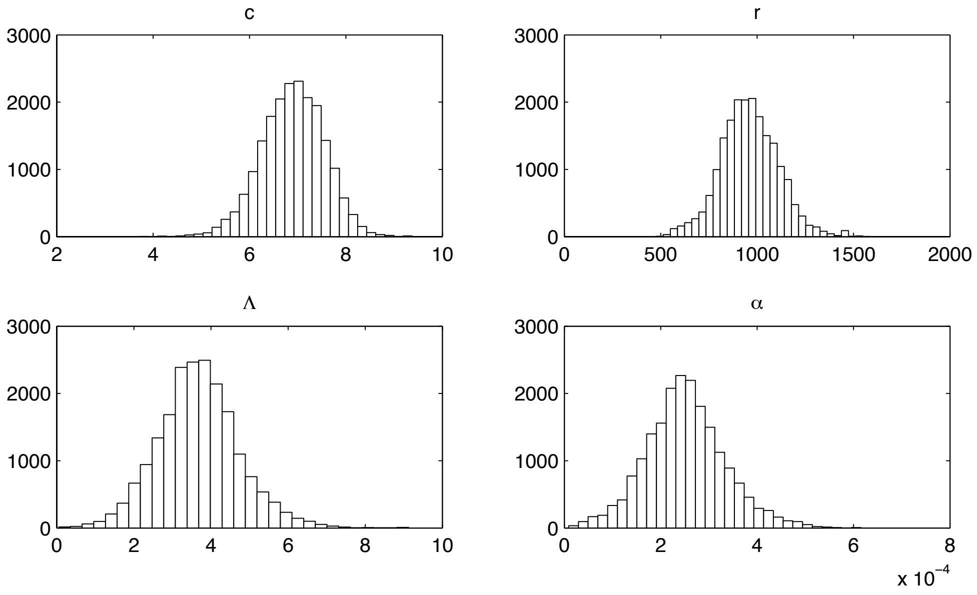

Supplement: Supplementary file 1 — Supplementary Material [file 41598_2018_33570_MOESM1_ESM.pdf]
